# Physiological and Psychological Effects of Treadmill Overtraining Implementation

**DOI:** 10.3390/biology10060515

**Published:** 2021-06-10

**Authors:** Yi Chung, Yi-Ting Hsiao, Wen-Ching Huang

**Affiliations:** 1 College of Human Development and Health, National Taipei University of Nursing and Health Sciences, Taipei 11219, Taiwan; chungyi@ntunhs.edu.tw; 2 Department of Exercise and Health Science, National Taipei University of Nursing and Health Sciences, Taipei 11219, Taiwan; 063214111@ntunhs.edu.tw; 3 Graduate Institute of Metabolism and Obesity Sciences, Taipei Medical University, Taipei 11031, Taiwan

**Keywords:** overtraining, treadmill, inflammation, animal model, performance

## Abstract

**Simple Summary:**

Overtraining occurs when an imbalance between training stress and recovery exists, and it is prevalent in athletes, soldiers, physical education, and health education undergraduates as well as a number of female and male adolescents. Despite a broad body of evidence concerning physiological and psychological correlates of this syndrome, the pathomechanisms of overtraining are still poorly understood. This illustrates the need to establish animal models of this disorder. This article outlines and discusses physiological and psychological effects of the current established overtraining model, based on an eight-week exhaustive treadmill exercise that reveals the involvement of imbalanced energy expenditure, exacerbated inflammatory response, increased intestinal permeability, and anxiety status in the development and onset of overtraining. This study highlights the maladaptation of overtraining and provides an animal model to determine the effectiveness of possible strategies, including nutrition and monitoring, for treatment and prevention of overtraining syndromes in future studies.

**Abstract:**

Overtraining in athletes usually causes profound and lasting deleterious effects on the maintenance of health and exercise capacity. Here, we established an overtraining animal model to investigate the physiological modulation for future strategic applications in vivo. We subjected C57BL/6 mice to exhaustive treadmill exercises daily for 8 weeks (the exhaustive exercise group). Next, the physiological and psychological outcomes were compared with the regular exercise and sedentary groups. Outcome measures included growth, glucose tolerance, exercise metabolism profiles, cytokine levels, intestinal tight junction gene expression, and psychological behavioral changes. Our results revealed that overtraining negatively affected the physiological and psychological changes in the current model. The exhaustive exercise group exhibited significantly lower endurance performance and imbalanced energy expenditure, causing a decrease in body fat mass and slowing down the growth curve. In addition, the inflammatory cytokines (tumor necrosis factor-alpha, interleukin-6, and interleukin-1β) and immune cells (neutrophils and monocytes) were significantly elevated after successive exhaustive exercise interventions. Furthermore, overtraining-induced stress resulted in increased anxiety status and decreased food intake. Our findings reinforce the idea that an imbalance between exercise and recovery can impair health and performance maintenance after overtraining. This study highlights the maladaptation of overtraining and provides an animal model to determine the effectiveness of possible strategies, including nutrition and monitoring, for treatment and prevention of overtraining syndromes in future studies.

## 1. Introduction

Striking a balance between training stress and recovery is critical for inducing adaptations to enhance athletic performance as the theory of supercompensation. However, continuously high training loads with inadequate recovery can cause fatigue accumulation, eventually resulting in overtraining [1]. Prolonged exposure to such training may lead to overtraining syndrome, which causes reduced performance and fatigue even after adequate resting and has no standardized diagnostic criteria [2]. Overtraining syndrome involves alterations in parasympathetic and sympathetic effects, such as fatigue, depression, low motivation, bradycardia, insomnia, irritability, agitation, and anorexia. In a survey involving athletes, approximately 30 and 60% of nonelite endurance athletes and elite athletes, respectively, were found to overtrain [3].

The lifetime prevalence of overtraining symptoms among athletes is 7–20%, and 30–100% of athletes report symptoms of overtraining after intense training [4]. Overtraining depends on individual differences and capabilities, so coaches and athletes are often unable to accurately determine whether overtraining occurs, which delays the immediate response and causes a long-term decrement in performance. In other applied settings, such as the military, soldiers are trained to withstand a high load of physical and psychological stress and thus can develop overtraining syndrome during either training courses or military operations. The prevention of overtraining syndrome can reduce the number of injuries and illnesses, leading to a more available workforce and an extended operational career, which represents a costly investment [5]. Therefore, it is critical to identify appropriate indicators that can effectively measure the risk level of training, and the possible hypotheses of overtraining syndrome etiology included glycogen, central fatigue, glutamine, oxidative stress, autonomic nervous system, hypothalamic, and cytokine hypotheses [6].

Overtraining may induce musculoskeletal injuries, cardiac arrhythmias, and biochemical and histological changes [7]. The cytokine hypothesis [8] considers that overtraining induces musculoskeletal trauma, increasing the production and release of proinflammatory cytokines, mainly tumor necrosis factor-alpha (TNF-α), interleukin 1-beta (IL-1β), and interleukin-6 (IL-6), which interact with various organic systems and trigger most of the signs and symptoms associated with performance decline [9]. Marked responses were also observed in hormone levels (cortisol, testosterone: cortisol ratio, and catecholamines) [10,11,12] as well as in blood biomarkers and muscle damage markers (lactate and creatine kinase (CK), respectively) [13]. However, these indicators of overtraining remain unclear due to the variability of the results, so these theories cannot be corroborated. Moreover, overtraining is not only related to a single training load but also has more reciprocal effects with physical, mental, and social factors [4]. Research has indicated detrimental effects of overtraining on athletes’ mental health, including increased depression, low motivation, anger, and eating disorders. Moreover, overtraining is demonstrated to cause burnout in up to 10% of athletes, which has been exhibited to affect their mood, self-esteem, and confidence and cause depression [14].

Long-term running at a challenging pace could result in small intestinal damage and increase intestinal permeability, but it is not correlated with the development of gastrointestinal symptoms during running [15]. In a meta-analysis study, a single bout of exercise also showed an increase in gut damage and gut permeability of healthy participants, which was even worse in hot environments [16]. The adaption of acute and chronic exercise intervention could affect the gut permeability and integrity through a number of mechanisms, including microbiota, inflammation, metabolites, antioxidant defense, and splanchnic blood flow [17]. Moreover, excessive exercise also elucidated the deleterious effects on immunity and substance and energy metabolism as well as gut microbial diversity by consecutive high-intensity swimming interventions [7]. Besides, the tight junction proteins, including Occludin, Claudin-1, and zona occludens (ZO)-1, are crucial for intestinal epithelial barrier integrity with disease development and pathological implications [18]. Therefore, the intestinal permeability and integrity could play critical roles for physiological modulation through microbiota and multiple physiological axes.

In this study, we developed an overtraining model of C57BL/6 mice by using a consecutive exhaustive treadmill exercise protocol, directly associated with physical fitness factors. Next, we investigated the effects of overtraining implementation on physiological parameters, including growth, glucose tolerance, lactate profiles, plasma cytokine levels, and intestinal tight junction gene expression, as well as on psychological aspects, such as behavioral changes, in the current animal model. We hypothesized that exhaustive exercise could exert deleterious effects on physiological and psychological conditions, and the energy metabolism, growth curve, inflammation, behaviors, intestinal permeability, and exercise performance could also be affected in the present treadmill exercise-induced overtraining mouse model.

## 2. Materials and Methods

### 2.1. Experimental Design

**Figure 1:**
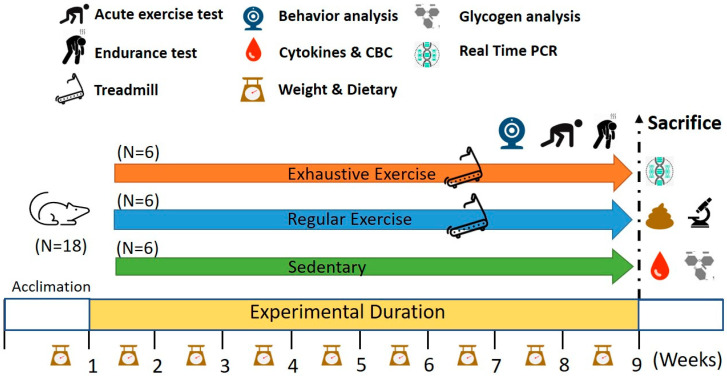
Experimental designs for the effects of regular exercise and exhaustive exercise murine model. The animals were randomly assigned to the indicated three groups (sedentary, regular exercise, and exhaustive exercise). The overtraining condition was induced using consecutively exhaustive running protocols. The physical fitness and related assessments were analyzed within the test duration and after sacrifice.

Specific-pathogen-free C57BL/6 mice (6 weeks old), purchased from BioLASCO (Yilan, Taiwan), were applied to the present study. The standard chow diet (No. 5001; PMI Nutrition International, Brentwood, MO, USA) and sterilized water were provided ad libitum during the experiment. The environment was maintained on a 12 h light/dark cycle at 24 ± 2 °C and 50–60% humidity, and the veterinarian monitored the behavior and disease status of the animals daily. After 1 week acclimation to the environment and diet, the animals were allocated into three groups (sedentary, regular exercise, and exhaustive exercise) based on their initial endurance capacities. To examine the initial endurance capacity before assigning groups, the exhaustive exercise treadmill protocol (in Section 2.3) was performed before and after the experiment. The endurance capacity was measured by the exhaustive swimming time. The body weight and endurance capacities demonstrated non-significant differences between groups at the beginning of the experiment. The different treadmill protocols were applied to indicated groups for 8 weeks duration (Figure 1). After the exercise intervention, their endurance capacities were evaluated by measuring exhaustive treadmill exercise, and exercise-associated biochemical indexes were also assessed in mice undergoing acute exercise challenges (20 min of treadmill running with an intensity of 18 m/min without any inclination). The diet was recorded for energy uptake references. Moreover, their behavior (psychological status in Section 2.7) was analyzed, and physiological effects were measured using the following parameters: the blood and relevant tissues, complete blood count (CBC), body composition, glycogen, and exercise-associated metabolites after acute exercise. The intestinal tight junction genes were also measured by real-time PCR. The evaluation of behavior, exhaustive endurance, and acute exercise was performed at the end of the study with at least 3 days interval. The experiment methods and procedures were reviewed and approved by the Institutional Animal Care and Use Committee (IACUC) with protocol number LAC-2020-0055 in Taipei Medical University.

### 2.2. Aerobic Treadmill Exercise Training

The mice in the regular exercise and exhaustive exercise groups underwent aerobic exercise training on a motor-driven treadmill (model 47,300 Treadmill; Ugo Basile, Gemonio, Italy) for 8 weeks, and their motivation was maintained through electric/air-puff/agitation shocks and physical prodding under veterinarian surveillance. All mice were familiarized with treadmill running at a speed intensity of 10 m/min for 10 min for 3 consecutive days prior to the training protocol. The regular exercise protocol was set at a speed of 15 m/min and followed by 18 m/min for 5 and 25 min, respectively, per day. The regular exercise training was performed on 3 nonconsecutive days per week for 8 weeks. An exhaustive exercise protocol was implemented in the exhaustive exercise group once per day for 4 weeks and increased to twice per day for the remaining 4 weeks. The first exhaustive training session was implemented at 08:00 a.m., and then the mice were provided a 4 h rest before the next exhaustive training session. 

### 2.3. Exhaustive Exercise Protocol and Endurance Capacities

In the current study, we applied and modified the incremental load test from previous studies [19] as a daily exhaustive exercise protocol in the exhaustive exercise group. The initial intensity of this test was 15 m/min at a 5% slope inclination, with increments of 3 m/min every 10 min until exhaustion, which was also defined as endurance capacities from beginning to exhaustion. The exhaustive fatigue behaviors were considered as spending 10 consecutive seconds in the fatigue zone, defined as the region including the proximal treadmill belt within approximately one body length of the shock grid. Both the electric shock (1 Hz and 0.1 mA) and air-puff were applied to the animal during acclimation to learn running behaviors, and the stimulus of the electric shock was gradually replaced by air-puff and pencil agitation in the first 2 weeks to prevent potential psychological and behavioral effects.

### 2.4. Peripheral Fatigue Biochemical Variables after Acute Exercise and Routine Blood Examination

Peripheral fatigue during exercise can be assessed using biochemical indexes for energy, stress, and metabolism. The acute exercise protocol included 20 min of treadmill running with an intensity of 18 m/min without any inclination, and blood was collected through submandibular venipuncture at the beginning and immediately after 20 min of running for the assessment of lactate levels and its production rate during exercise. In addition, the ammonia, lactate dehydrogenase (LDH), and CK were also analyzed immediately after exercise. The blood samples were assessed with an autoanalyzer (Hitachi 7060; Hitachi, Tokyo, Japan). The blood was collected into microtubes containing the anticoagulant K2-EDTA for CBC analysis (ADVIA 2010, Bayer, NY, USA).

### 2.5. Cytokine Analysis

After the mice were subjected to CO_2_ asphyxiation euthanasia, blood was immediately withdrawn through cardiac puncture, and the serum was isolated through centrifugation at 1000× *g* for 12 min at 4 °C after complete clotting for cytokine analysis. Serum samples (50–100 μL) were quantified for TNF-α, IL-1 beta, and IL-6 levels by using specific ELISA kits (Invitrogen, Carlsbad, CA, USA) following the manufacturer’s instructions and measured using a spectrophotometer (EnSpire Multimode Plate Reader, PerkinElmer, CA, USA).

### 2.6. Behavioral Assessment Using Open Field and Elevated Plus Maze Tests

Mice behaviors were evaluated using open field and elevated plus maze tests for locomotor activity levels and anxiety status after the indicated training interventions. The elevated plus maze test was performed 4 h after the open field test. The detailed procedures and apparatuses were described in our previous study [20], and the behavioral tests of the indicated behavior videos were analyzed using Actual Track software (Actual Analytics, Edinburgh, UK) for 10 and 5 min, respectively. The physiological indexes of anxiety were traveling distance and central area time percentage for the open field test and entry frequencies and duration of open/close arms for the elevated plus maze test. The feces and urine in the apparatus were removed, and the apparatus was cleaned with 75% ethanol before the next mouse was placed in.

### 2.7. Glucose Tolerance Test

Mice were orally administered 1.0 g of glucose per kg of body weight after 12 h fasting. The whole blood (0.6 μL) from the tail was sampled at the indicated time points by using a glucometer (Accu-Chek^®^; Roche, Taipei, Taiwan) for determination of the plasma glucose concentration [21]. 

### 2.8. Body Composition, Histology, and Glycogen Analysis

The animals received the indicated training and were provided a sufficient diet ad libitum one day before euthanasia. After euthanasia, the critical tissues and organs (heart, liver, kidney, muscle, and epididymal fat pad) were accurately pruned and weighed for body composition assessment. Next, the organs were preserved in 10% formalin and paraffin embedded for histological examination; 4-μm-thick sections were obtained from paraffin blocks and immersed in xylene and alcohol, followed by staining with hematoxylin–eosin. Moreover, a part of the muscle and liver samples was maintained in liquid nitrogen for glycogen content analysis. The analysis protocol of glycogen content was slightly modified from a previous study [22].

### 2.9. RNA Extraction and Real-Time Quantitative Polymerase Chain Reaction

The proximal colon was immediately immersed in RNA Save (Biological Industries, Beit Haemek, kibbutz, Israel) and stored at −80 °C after euthanasia for further analysis. The samples were extracted using an EZ-RNA II total RNA isolation kit (Biological Industries Beit Haemek, Israel). Next, 1 μg of RNA per 20 μL of reaction volume was reverse transcribed to cDNA using ToolsQuant II Fast RT Kit (BIOTOOLS, Taipei, Taiwan). The quantitative polymerase chain reaction (qPCR) reactions were conducted using TOOLS 2X SYBR qPCR Mix (BIOTOOLS) in LightCycler 480 (Roche, Switzerland). The cDNA template (2 μL of samples in a total volume of 20 μL per reaction) was analyzed for the tight junction genes (*Occludin*, *Claudin-1*, and *ZO-1*). The tight junction gene primers (*Occludin*: 5′ AGGTCTGGCGACATTAGTGG3′ and 5′CGTGGTGTTGGGTAAGAGGT3′; *Claudin-1*: 5′TTGAAAGTCCACCTCCTTACAGA3′ and 5′CCGGATAAAAAGAGTACGCTGG3′; and *ZO-1*: 5′GCACCATGCCTAAAGCTGTC3′ and 5′ACTCAACACACCACCATTGC3′) and internal control gene primers (*GAPHD*: 5′GTTGTCTCCTGCGACTTCA3′ and 5′GGTGGTCCAGGGTTTCTTA3′) were applied to this qPCR for relative expression with different treatments. The primer specificity was validated by a dissociation (melting) curve after the process of real-time PCR. The indicated genes (*Occludin*, *Claudin-1*, *ZO-1,* and *GAPDH*) were calculated by the threshold cycle (Ct) value, and the relative mRNA expression level was further calibrated by *GAPDH* expression.

### 2.10. Statistical Analysis

The Data in the current study were represented as the mean ± standard deviation. Significant differences of intergroup with the physical activities, biochemistry, CBC, body composition, cytokines, and glycogen content were analyzed by using one-way analysis of variance. Mixed two-way analysis of variance was applied to the growth curve and glucose tolerance test. The multiple comparisons between groups were verified by the *post hoc* Tukey test. Data were analyzed by SPSS Statistics 19.0 (IBM, New York, NY, USA) and considered statistically significant when the *p* value was less than 0.05. In figures and tables, the significant differences between treatments were represented by the compact letter display method with various subscript Roman letters (a, b, c, d).

## 3. Results

### 3.1. Effects of Exercise Training Intervention on Growth Curve and Body Composition

**Figure 2:**
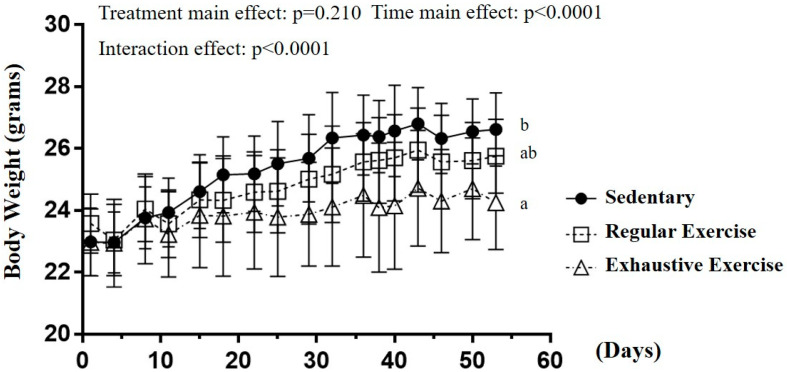
The growth curve with eight-week indicated exercise intervention. Treatments with different letters (a, b) differ significantly at *p* < 0.05 by using one-way analysis of variance.

The body weight in sedentary, regular exercise, and exhaustive exercise groups before training initiation was 23.0 ± 1.1, 23.6 ± 1.0, and 23.0 ± 1.1 g, respectively, and the differences were not significant (F(2, 15) = 0.677, *p* = 0.523). After 8 weeks of training interventions, the time main effect and interaction effect were significantly different (F(16, 240) = 71.62, *p* < 0.0001; F(32, 240) = 5.832, *p* < 0.0001; Figure 2). For the simple main effect, a significant difference in body weight was observed among the groups (F(2, 15) = 4.99, *p* = 0.022) at the end of the study. The body weight in the exhaustive group was significantly lower than that in the sedentary group (*p* = 0.007) but that in the regular exercise group was not significantly different from the other two groups. The different training intensities could indicate the different effects on the growth curve, and possible physiological effects were evaluated.

The body composition was evaluated using the weights of individual tissue and organs, and the diet was recorded as quantity/volume per day (Table 1). The epididymal fat pad mass was significantly different among the groups (F(2, 15) = 8.74, *p* = 0.003), with significantly lower mass in the exhaustive exercise group than in the sedentary and regular exercise groups. No significant intergroup differences were observed in other organs. A significant difference among groups was observed in diet (F(2, 103) = 6.59, *p* = 0.002) but not in water intake (F(2, 103) = 2.14, *p* = 0.123). The dietary intake of the exhaustive exercise group significantly decreased compared with the sedentary and regular exercise groups.

### 3.2. Effects of Exercise Training Intervention on the Endurance Capacity Profile

**Figure 3:**
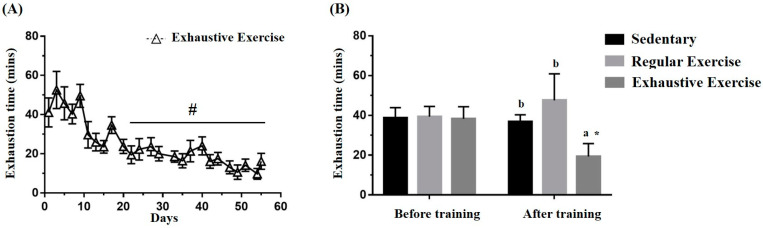
Effects of the eight-week training intervention on endurance performance. (**A**) The endurance capacity profile in the exhaustive exercise group throughout the study period. (**B**) The endurance capacities in all groups before and after the study. The data are mean ± standard deviation. Columns with different letters (a, b) are significantly different at *p* < 0.05 between groups by using one-way ANOVA. * *p* < 0.05 within the group and # *p* < 0.05 compared with day 1 exercise capacity by paired *t* test.

The exhaustive exercise protocol was implemented daily, and endurance capacity was recorded. Figure 3A illustrates the representative 3 days/week for conciseness in the trend of the exhaustive exercise group. The exhaustive exercise group also exhibited a significant difference among the endurance capacity profiles as obtained using repeated one-way ANOVA (F(24, 120) = 13.02, *p* < 0.0001), and the endurance capacities exhibited a significant decrement from the 4th to 8th week compared with day 1. The endurance capacities of the mice were assessed for group assignment reference, and Figure 3B demonstrates no significant differences in the endurance capacities among groups before training (F(2, 15) = 0.06, *p* = 0.942). After the eight-week indicated training, endurance was significantly different between groups (F(2, 15) = 13.39, *p* < 0.0001). The endurance capacity of the exhaustive exercise group was significantly lower than that of the sedentary and regular exercise groups (*p* = 0.001 and *p* < 0.0001) and that of the regular exercise group was higher than that of the sedentary group, with slight significance (*p* = 0.052). Significance was also observed within the exhaustive exercise group but not in the other groups.

### 3.3. Effects of Exercise Training Intervention on Glucose Tolerance

**Figure 4:**
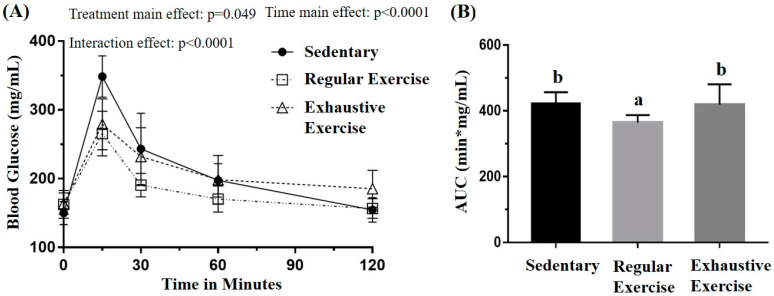
Effects of the eight-week training intervention on glucose tolerance (**A**) and area under the curve (AUC) (**B**). The oral glucose tolerance test was performed at the end of the study, and blood samples were collected at 0, 15, 60, 90, and 120 min after the oral dose of 1 g/kg glucose. Data are represented as mean ± standard deviation, and the columns with different letters (a, b) are significantly different between groups (*p* < 0.05) by using one-way ANOVA.

The glucose tolerance was assessed using the oral glucose tolerance test to evaluate the glucose homeostasis in the three groups, with the blood sampled from the tail vein at the indicated time points (Figure 4A). The oral glucose tolerance profiles indicated the main effect for treatment (F(2, 15) = 3.75, *p* = 0.048) and time (F(4, 60) = 104.63, *p* < 0.0001) as well as a significant treatment × time interaction (F(8, 60) = 5.884, *p* < 0.0001) by using mixed two-way ANOVA. Further analysis of the simple main effect revealed a significant difference among groups at 15 and 120 min (F(2, 15) = 10.63, *p* = 0.001 and F(2, 15) = 4.30, *p* = 0.033, respectively). The glucose levels in the regular exercise and exhaustive exercise groups were both significantly lower than that in the sedentary group (*p* = 0.002 and 0.007, respectively) at 15 min, and, at 120 min, the glucose level in the exhaustive exercise group was significantly higher than those in the other groups (*p* < 0.05). The area under the curve was also significant among treatments, with that of the regular exercise group being significantly lower than that of the other groups (*p* < 0.05).

### 3.4. Effects of Exercise Training Intervention on Fatigue-Associated Biochemistry

**Figure 5:**
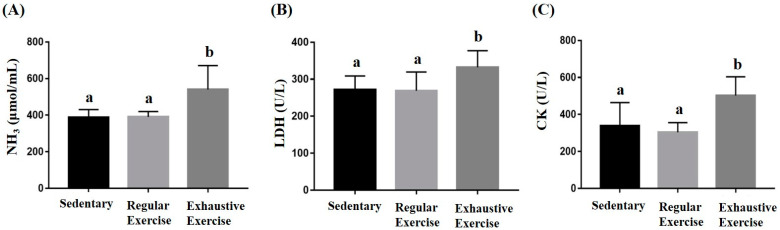
Effect of the eight-week training intervention on NH_3_ (**A**), LDH (**B**), and CK (**C**) levels after an acute exercise challenge (20 min treadmill). Blood was immediately sampled after the exercise for biochemical assessments. Data are represented as mean ± standard deviation, and the columns with different letters (a, b) indicate significant difference at *p* < 0.05.

Peripheral fatigue resulting from energy demand, oxidative stress, lactate metabolism during exercise, and the intensity and duration of exercise could be reflected by the associated biochemical indexes (lactate, ammonia, CK, and LDH). In the metabolite and injury indexes, ammonia, LDH, and CK were significantly different among the groups (F(2, 15) = 6.88, *p* = 0.008; F(2, 15) = 3.93, *p* = 0.042; and F(2, 15) = 6.95, *p* = 0.007, respectively). The exhaustive exercise group exhibited significantly higher levels of ammonia, CK, and LDH than the other two groups (Figure 5). Higher lactate levels during exercise indicated energy production from the anaerobic glycolysis system, which may be considered a less efficient energy system. The lactate representing the energy demands and energy metabolites was assessed during the acute exercise challenge (Table 2). The lactate levels before and after running revealed significant differences among groups (F(2, 15) = 6.58, *p* = 0.009 and F(2, 15) = 11.91, *p* = 0.001, respectively). The exhaustive exercise group had a significantly higher lactate level and lactate production rate than the other two groups (*p* = 0.027 and 0.04, respectively).

### 3.5. Effects of the Exercise Training Intervention on Cytokines and Glycogen

**Figure 6:**
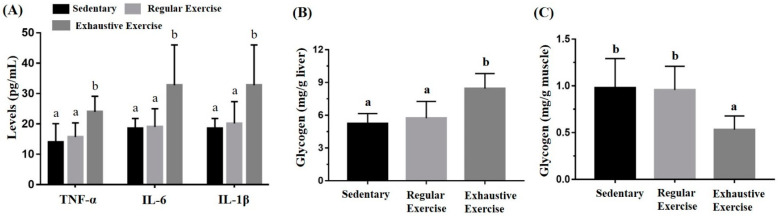
Effects of the eight-week training intervention on plasma cytokine expressions (**A**), liver glycogen (**B**), and muscle glycogen (**C**). The serum and tissue samples were collected at the end of the study for cytokine and glycogen analyses. Data are represented as mean ± standard deviation, and the columns with different letters (a, b) represent a significant difference between groups (*p* < 0.05) by using one-way ANOVA.

The liver and muscle (gastrocnemius and soleus) glycogen content was determined through colorimetric assessment of glycogen–iodine complexes. Significant differences in liver and muscle glycogen content were observed (F(2, 15) = 9.20, *p* = 0.002 and F(2, 15) = 6.21, *p* = 0.011, respectively) among the groups (Figure 6B,C). Liver glycogen was significantly higher in the exhaustive exercise group than in the sedentary and regular exercise groups (*p* = 0.001 and 0.005, respectively), but no significant difference was observed between the sedentary and regular exercise groups. Muscular glycogen was significantly higher in the sedentary and regular exercise groups than in the exhaustive exercise group (*p* = 0.007 and 0.01, respectively). TNF-α, IL-6, and IL-1β levels were also significantly different among the groups (F(2, 15) = 6.07, *p* = 0.012; F(2, 15) = 5.24, *p* = 0.019; and F(2, 15) = 4.55, *p* = 0.028, respectively). TNF-α, IL-6, and IL-1β levels were significantly higher in the exhaustive exercise group than in the sedentary and regular exercise groups (Figure 6A).

### 3.6. Effects of Exercise Training Intervention on CBC

In the CBC analysis, total white blood cell (WBC) count and differential WBC counts, including neutrophil, lymphocyte, and monocyte counts, were significantly different (F(2, 15) = 3.73, *p* = 0.042; F(2, 15) = 13.7, *p* < 0.0001; F(2, 15) = 17.0, *p* < 0.0001; and F(2, 15) = 7.85, *p* = 0.005, respectively). Compared with the sedentary and regular exercise groups, the exhaustive exercise group had significantly higher neutrophil and monocyte counts and a lower lymphocyte count. In addition, the platelet-to-lymphocyte ratio (PLR) and neutrophil-to-lymphocyte ratio (NLR) were significantly different among the groups (F(2, 15) = 8.71, *p* = 0.003 and F(2, 15) = 10.21, *p* = 0.002, respectively), being significantly higher in the exhaustive exercise group than in the other two groups (Table 3).

### 3.7. Effects of Exercise Training Intervention on Behavior Analysis

**Figure 7:**
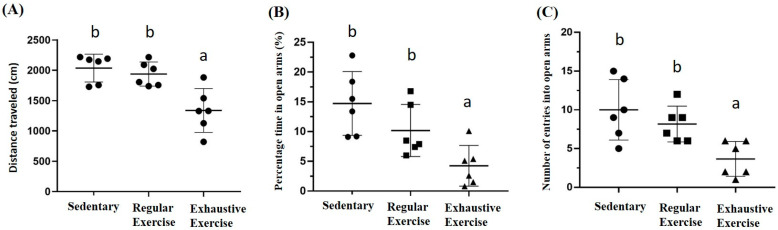
The effects of eight weeks indicated exercise training intervention on locomotor activity levels, and anxiety status after indicated training interventions. Locomotor activity levels (**A**), percentage of time spent in open arms (%) of the elevated plus maze (**B**), and number of entries into the open arms of the elevated plus maze (**C**) were analyzed by Actual Tract software. Data are represented as mean ± SD and the columns with different letters (a, b) represented the significant difference between groups (*p* < 0.05) by using one-way ANOVA.

The locomotor activity was represented by distance traveled in the open field area, and a significant difference was observed among groups (F(2, 15) = 11.6, *p* = 0.001). The exhaustive exercise group had significantly lower locomotor activity than the sedentary and regular exercise groups (*p* < 0.0001 and *p* = 0.002, respectively; Figure 7A). Anxiety in the elevated plus maze was assessed using the stay duration and frequency between closed and open arm regions. The duration and frequency between regions were significantly different among groups (F(2, 15) = 8.3, *p* = 0.004 and F(2, 15) = 7.5, *p* = 0.006, respectively), with significantly lower values in the exhaustive exercise group than in the sedentary and regular exercise groups (both *p* < 0.05; Figure 7B,C).

### 3.8. Effects of Exercise Training Intervention on Colon Tight Junction Genes

**Figure 8:**
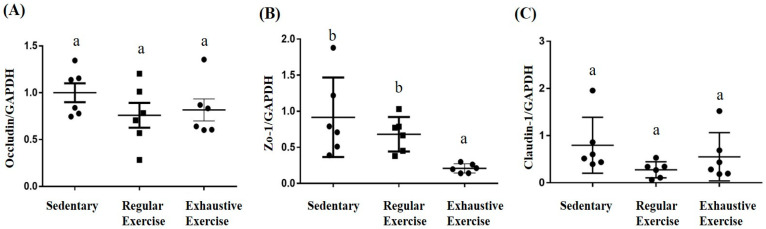
Effects of the eight-week training intervention on tight junction proteins in the proximal colon. Relative expression of *Occludin* (**A**), *ZO-1* (**B**), and *Claudin-1* (**C**) were analyzed using quantitative polymerase chain reaction and normalized by GAPDH. Data are represented as mean ± standard deviation, and columns with different letters (a, b) represent significant difference between groups (*p* < 0.05) by using one-way ANOVA.

In the proximal colon, a significant difference among groups was noted in terms of ZO-1 mRNA expression (F(2, 15) = 5.89, *p* = 0.013), with the levels being significantly lower in the exhaustive exercise group than in the sedentary and regular exercise groups (Figure 8B). The intergroup differences were not significant for *Occludin* and *Claudin-1* expression (F(2, 15) = 1.14, *p* = 0.345 and F(2, 15) = 1.9, *p* = 0.184, respectively).

## 4. Discussion

We developed an overtraining mouse model by subjecting mice to exhaustive treadmill exercise every day for 8 weeks; we observed a significant reduction in endurance performance compared with mice in the sedentary and the regular exercise groups. Furthermore, our results demonstrated that overtraining could cause deleterious physiological and psychological effects in the current established mouse model. The overtraining syndrome in the exhaustive exercise group significantly affected the growth curve, inflammation, exercise energy metabolism, and behaviors; these changes can also be observed in athletes.

The alterations from weeks 0 to 8 for low food intake and body weight gain observed in mice subjected to overtraining agree with previous studies [23,24,25]. Overtraining induces transitory inflammation, which may cause reduced food intake and body weight gain in overtrained mice [25]. Excessive training can inhibit the appetite, which affects the maintenance of proper blood glucose levels for specific organs, for example, the brain [9]. Accordingly, the observed decrease in body weight may be due to hypermetabolism and proteolysis as a result of high training loads and insufficient recovery periods [26].

To elucidate whether overtraining was associated with disturbed metabolic function, we performed OGTT at the end of the study. Our findings revealed that the regular exercise group had better glucose control than both the control and exhaustive exercise groups, supporting the notion that regular exercise can improve glucose tolerance. Regular exercise is known to be associated with improved glucose tolerance, as well as insulin sensitivity, and hemoglobin A1c while muscle contraction stimulates glucose transport by insulin-independent mechanisms [27]. The adaptation of regular exercise may facilitate a fairly rapid rate of glucose uptake by the tissues, mainly muscle, resulting in normal or improved glucose tolerance even with low insulin levels [28]. Therefore, after regular exercise with appropriate intensity and duration, the body could handle the exercise stress, and, consequently, adaptation occurred. This adaptive effect may regulate the health beneficial effects [29]. 

Glucose homeostasis is regulated by the liver, which maintains the uptake and storage of glucose through glycogenesis as well as its release through glycogenolysis and gluconeogenesis [30]. The liver may play a key role in maintaining glucose homeostasis in the glucose transporter protein of the skeletal muscle in knockout mice [31], and glucose is partially shifted to the liver in rodents with skeletal muscle deletion of the glucose transporter protein [32]. Excessive training may impair the insulin signaling pathway in skeletal muscle without significant changes in the insulin tolerance test [33]. Taken together, these results reinforce the ideas that overtraining may be associated with glucose intolerance, the liver may act as a compensatory organ for glucose homeostasis [9] and improve major proteins involved in hepatic insulin signaling, which induces hepatic glycogen accumulation [23]. Correspondingly, our results revealed a significant increase in hepatic glycogen in the exhaustive exercise group, along with a decrease in muscle glycogen. Glycogen rapidly provides muscle cells with ATP, which display a high and rapidly shifting energy turnover in skeletal muscle [34]. The quantity of muscle glycogen storage can directly affect exercise performance [35] and is altered depending on the exercise intensity [36]. Repeated intense training could lead to delayed glycogen re-synthesis [37]. The glycogen hypothesis of overtraining syndrome states that exercise-induced muscle glycogen depletion is linked to decreased performance [38]. Accordingly, in the current study, muscle glycogen content was significantly lower in the exhaustive training group than in the other groups.

Levels of fatigue-related parameters, including lactate, ammonia, LDH, and muscle damage enzymes such as CK, increased in the exhaustive exercise group. Moreover, impairment of muscle glycogen restoration, another detrimental effect of exercise-induced muscle damage, is indicated by elevated plasma levels of CK and LDH [39]. These findings correspond to previous studies that increased LDH and CPK levels are attributed to exercise-induced muscle fiber damage, leading to muscle pain and fatigue, which can result in reduced performance and exercise-related injuries [39,40]. The exhaustive exercise may also cause oxidative stress and tissue damage. Citrate synthase, a vital enzyme in aerobic oxidation, was observed to be decreased in overtrained rats, making them more susceptible to oxidative stress [26]. Elevated lactate levels can reduce blood pH in terms of various physiological and biochemical side effects including glycolysis, as well as phosphofructokinase and calcium ion release from muscular contraction [41]. Therefore, high energy demand during exercise may activate anaerobic glycolysis, which results in increased lactate levels and eventually a decline in exercise performance, as was observed in our treadmill overtraining model. LDH is a specific enzyme found in red blood cells and muscle cells that can be used to evaluate the energy system under a variety of exercise conditions. It characterizes the degree of exercise intensity, muscle stiffness, fatigue recovery, and adaptation of metabolic function during energy metabolism, as well as excessive training and histological damage analysis [42,43]. High-intensity exercise damages the muscle cells, and cell permeability is increased, leading to LDH release and high blood LDH levels. The muscles are overloaded and influenced by tissue necrosis or cell membrane destruction, which increases blood LDH and CK concentrations. These biomarkers, lactate, CK, ammonia, inflammation cytokines (IL-6 and TNF-α), leukocyte, and oxidative stress were considered as part of the peripheral fatigue indexes to evaluate the physiological status during exercise [44]. The overtraining syndrome could be caused by not only fatigue accumulation but also other several factors, including psychological and/or social stressors and physiologic stress. Thus, we found the exhaustive exercise group with overtraining syndrome could also be observed by the significantly elevated peripheral fatigue biomarker and psychological behaviors in the current results.

CBC analysis revealed higher neutrophil and monocyte counts and a lower lymphocyte count in the exhaustive exercise group than in the sedentary and regular exercise groups, which may be due to the inflammatory response induced by tissue injury; these results were consistent with the hematologic parameter changes observed in runners after a marathon [45]. Our findings also agree with studies reporting that following prolonged or intense exercise, neutrophil and monocyte counts increase, whereas the lymphocyte count decreases, which causes an “open window” of immunodepression in long-distance runners and healthy adults [46,47]. Increased NLR may be a potential immune-inflammation marker for approaching overtraining [39]. Moreover, considering exercise-induced thrombocytosis, PLR might be a useful addition or alternative to NLR, as shown by increases in both markers following acute exercise in both healthy [48] and diseased populations [49]. Intriguingly, PLR values were changed more extremely by high-intensity exercise with values approximately twice those at rest [48]. One explanation for this might be the increase in platelets, mobilized by high intensity exercise, into the peripheral circulation [50]. The combination of decreased lymphocytes and increased neutrophils, monocytes, and platelets supports the theory that a compromised immune system underlies the pathophysiology of overtraining syndrome and related conditions, rendering mice susceptible to infection [7]. Furthermore, overtraining causes tissue injury and the release of trauma-related cytokines, which activate circulating neutrophils and monocytes to produce the inflammatory cytokines TNF-α, IL-1β, and IL-6 [8]. 

Previous studies from rodents support the connection between inflammatory cytokines and overtraining syndrome. A study showed that overtraining, induced in rats by subjecting them to 11 weeks of motorized treadmill running, led to reduced physical performance along with increased TNF-α and IL-6 levels as compared to sedentary control and moderately trained control groups [29]. Similarly, the increased TNF-α, IL-1β, and IL-6 levels were also found in male C57BL/6 mice after an eight-week excessive treadmill exercise [25] and in male Wistar rats after an 11-week excessive exercise [51]. The results in rodents support the idea that an imbalance between overload and recovery increases serum TNF-α, IL-1β, and IL-6 levels immediately after the overtraining period [9]. Several causes may underlie the decreased immune function associated with overtraining [39]. One mechanism may simply be the cumulative effects of repeated bouts of exhausting exercise with the resultant elevation of stress hormones (mainly glucocorticoids), leading to temporary immunosuppression [52]. In addition, complement activation occurs during exercise; a reduction in serum complement concentration with repeated bouts of exercise, especially when accompanied by muscle damage, could also contribute to reduced nonspecific immunity [53].

Upregulation of proinflammatory cytokines, including TNF-α, IL-6, and IL-1β signaling, disrupts tight junction proteins, resulting in enhanced intestinal permeability and downregulation of the expression of the tight junction protein ZO-1 [54], which is consistent with our finding that ZO-1 expression in the exhaustive exercise group was significantly lower than that in the sedentary and regular exercise groups. ZO-1 and Claudin-1 are vital tight junction proteins that form the tight junction seal [55], whereas Occludin helps maintain the tight junction complex [56]. Increased intestinal permeability allows endotoxins to enter the bloodstream, which can trigger local and systemic inflammatory responses [57]. Another perspective is that prolonged running may disrupt tight junction proteins through increased heat production in the intestinal wall coupled with ischemia/reperfusion stress [58]. Together, these findings suggest an inflammatory status leading to increased susceptibility to infections after overtraining.

Overtraining can also have negative effects on athletes’ mental health, such as increased depression, low motivation, anger, and eating disorders. It causes burnout in up to 10% of athletes, manifesting as impaired mood, low self-esteem, loss of confidence, and depression [14]. Behavioral and psychological changes in overtraining syndromes may result from inflammation cytokines [6]. The proinflammatory IL-1β and TNF-α cytokines could also interfere with the brain and lead to decreased appetite, sleep disturbance, and depression [8]. These support our behavioral results that the exhaustive exercise group may develop psychological symptoms, such as anxiety. Of several possible mechanisms, we proposed that the microbiota may play critical roles during overtraining through energy metabolism regulation, physiological regulation, and immune modulation and may even cause systemic effects. It may be modulated by multiple physiological axes, including the gut–brain, gut–muscle, and hypothalamic–pituitary–adrenal axes. In addition, it has been shown that high intensity exercise with acute sleep disturbance could cause the tissue injury and inflammation but not anxiety and memory decrease [20]. Therefore, the circadian rhythm, possibly interfered with by treadmill training, could also be an important factor for physiological and psychological effects in the onset of overtraining. However, this hypothesis needs to be further explored and verified in future clinical and animal experiments.

## 5. Conclusions

Appropriate exercise intensity can improve exercise capacity through physiological adaptations, but excessive exercise without sufficient rest can impair health maintenance and exercise performance. Other than adequate rest, no effective intervention exists to counteract overtraining syndromes or accelerate recovery. However, anti-inflammatory compounds may neutralize the elevated chronic inflammation in the muscles of athletes with overtraining, although further research is required to validate the effectiveness of these strategies. The athletes who suffered or were conditioned in overtraining would be the main target population for clinical application. In terms of research ethics, directly using human trials to investigate the physiological and psychological effects on the processes of overtraining would pose potential risks to health. Therefore, overtraining should be studied in a well-established animal model, and the possible mechanisms and effects could be further investigated and validated. The present study highlighted the maladaptation of overtraining and developed an overtrained animal model, which may be used to elucidate the effectiveness of future strategies, including nutrition intervention, alternative medicines, and physiotherapy, for amelioration, prevention, and monitoring of overtraining syndromes.

## Figures and Tables

**Table 1:** Effects of consecutive exhaustive exercise intervention on body composition and diet.

Characteristic	Sedentary	Exercise	Exhaustion
Liver (g)	1.11 ± 0.12	1.10 ± 0.10	1.03 ± 0.07
Muscle (g)	0.31 ± 0.02	0.30 ± 0.02	0.28 ± 0.03
Kidney (g)	0.28 ± 0.01	0.30 ± 0.01	0.29 ± 0.03
Heart (g)	0.14 ± 0.02	0.15 ± 0.02	0.14 ± 0.03
EFP (g)	0.33± 0.06 ^b^	0.27± 0.03 ^a^	0.24 ± 0.01 ^a^
Diet (g/mouse/day)	4.49 ± 0.59 ^b^	4.32 ± 0.64 ^b^	3.89 ± 0.87 ^a^
Water (mL/mouse/day)	7.14 ± 1.02	7.00 ± 2.22	8.08 ± 3.37

Exercise and Exhaustion represent the regular exercise and exhaustive exercise groups, respectively. Values in the same row with different superscript letters (a, b) differ significantly, *p* < 0.05, by using one-way analysis of variance. Muscle: gastrocnemius and soleus; EFP: epididymal fat pad.

**Table 2:** Effects of consecutive exhaustive exercise intervention on lactate metabolite profiles after an acute exercise challenge.

Time Point	Sedentary	Exercise	Exhaustion
	Lactate (mmol/L)		
Before running (A)	2.48 ± 0.16 ^a^	2.45 ± 0.26 ^a^	2.93 ± 0.33 ^b^
After running (B)	3.78 ± 0.23 ^a^	3.77 ± 0.61 ^a^	5.83 ± 1.31 ^b^
	Lactate production rate		
Production rate = B/A	1.53 ± 0.09 ^a^	1.56 ± 0.32 ^a^	1.98 ± 0.32 ^b^

Exercise and Exhaustion represent the regular exercise and exhaustive exercise groups, respectively. Lactate was measured before and after the acute exercise. In one-way ANOVA, the treatment with different superscript letters (a, b) in the same row differ significantly when *p* < 0.05.

**Table 3:** Effects of consecutive exhaustive exercise intervention on complete blood count analysis.

Parameter	Sedentary	Exercise	Exhaustion
WBC (10^3^/μL)	4.4 ± 1.1 ^b^	3.6 ± 0.7 ^ab^	3.3 ± 0.8 ^a^
Neu (%)	15.0 ± 2.8 ^a^	12.5± 2.9 ^a^	28.0 ± 8.6 ^b^
Lym (%)	84.5 ± 3.0 ^b^	86.7 ± 2.8 ^b^	69.5 ± 8.7 ^a^
Mono (%)	0.5 ± 0.8 ^a^	0.9 ± 1.2 ^a^	2.7 ± 1.0 ^b^
Eosi (%)	0.10 ± 0.1	0.09± 0.1	0.09 ± 0.1
Baso (%)	0.11 ± 0.1	0.12 ± 0.1	0.11 ± 0.2
RBC (million/μL)	10.2 ± 0.4	10.0 ± 0.2	10.3 ± 0.3
Hb (g/dL)	15.6 ± 0.4	15.4 ± 0.4	15.5 ± 0.6
Platelet (10^3^/μL)	1077 ± 65 ^ab^	1025 ± 77 ^a^	1149 ± 86 ^b^
PLR	0.31 ± 0.1 ^a^	0.34 ± 0.1 ^a^	0.53 ± 0.1 ^b^
NLR	0.18 ± 0.04 ^a^	0.14 ± 0.04 ^a^	0.42 ± 0.2 ^b^

Exercise and Exhaustion represent the regular exercise and exhaustive exercise groups, respectively. Data are expressed as mean ± standard deviation. Values in the same row with different superscript letters (a, b) differ significantly; *p* < 0.05, by using one-way ANOVA. WBC, white blood cell count; Neu, neutrophil count; Lym, lymphocyte count; Mono, monocyte count; Eosi, eosinophil count; Baso, basophil count; RBC, red blood count; Hb, hemoglobin level; PLR, platelet/lymphocyte ratio; NLR, neutrophil/lymphocyte ratio.

## Data Availability

The data presented in this study are available upon request from the corresponding authors.

## References

[1] Grandou C., Wallace L., Impellizzeri F.M., Allen N.G., Coutts A.J. (2020). Overtraining in Resistance Exercise: An Exploratory Systematic Review and Methodological Appraisal of the Literature. Sports Med..

[2] Cadegiani F.A., da Silva P.H.L., Abrao T.C.P., Kater C.E. (2020). Diagnosis of Overtraining Syndrome: Results of the Endocrine and Metabolic Responses on Overtraining Syndrome Study: EROS-DIAGNOSIS. J. Sports Med..

[3] Meeusen R., Duclos M., Foster C., Fry A., Gleeson M., Nieman D., Raglin J., Rietjens G., Steinacker J., Urhausen A. (2013). Prevention, diagnosis, and treatment of the overtraining syndrome: Joint consensus statement of the European College of Sport Science and the American College of Sports Medicine. Med. Sci. Sports Exerc..

[4] Matos N.F., Winsley R.J., Williams C.A. (2011). Prevalence of nonfunctional overreaching/overtraining in young English athletes. Med. Sci. Sports Exerc..

[5] Vrijkotte S., Roelands B., Pattyn N., Meeusen R. (2019). The Overtraining Syndrome in Soldiers: Insights from the Sports Domain. Mil. Med..

[6] Kreher J.B., Schwartz J.B. (2012). Overtraining syndrome: A practical guide. Sports Health.

[7] Yuan X., Xu S., Huang H., Liang J., Wu Y., Li C., Yuan H., Zhao X., Lai X., Hou S. (2018). Influence of excessive exercise on immunity, metabolism, and gut microbial diversity in an overtraining mice model. Scand. J. Med. Sci. Sports.

[8] Smith L.L. (2000). Cytokine hypothesis of overtraining: A physiological adaptation to excessive stress?. Med. Sci. Sports Exerc..

[9] da Rocha A.L., Pinto A.P., Kohama E.B., Pauli J.R., de Moura L.P., Cintra D.E., Ropelle E.R., da Silva A.S.R. (2019). The proinflammatory effects of chronic excessive exercise. Cytokine.

[10] Fry A.C., Kraemer W.J., Ramsey L.T. (1998). Pituitary-adrenal-gonadal responses to high-intensity resistance exercise overtraining. J. Appl. Physiol..

[11] Fry A.C., Kraemer W.J., Van Borselen F., Lynch J.M., Triplett N.T., Koziris L.P., Fleck S.J. (1994). Catecholamine responses to short-term high-intensity resistance exercise overtraining. J. Appl. Physiol..

[12] Sterczala A.J., Fry A.C., Chiu L.Z.F., Schilling B.K., Weiss L.W., Nicoll J.X. (2017). β2-adrenergic receptor maladaptations to high power resistance exercise overreaching. Hum. Physiol..

[13] Fry A.C., Kraemer W.J., van Borselen F., Lynch J.M., Marsit J.L., Roy E.P., Triplett N.T., Knuttgen H.G. (1994). Performance decrements with high-intensity resistance exercise overtraining. Med. Sci. Sports Exerc..

[14] Hughes L., Leavey G. (2012). Setting the bar: Athletes and vulnerability to mental illness. Br. J. Psychiatry.

[15] Karhu E., Forsgard R.A., Alanko L., Alfthan H., Pussinen P., Hamalainen E., Korpela R. (2017). Exercise and gastrointestinal symptoms: Running-induced changes in intestinal permeability and markers of gastrointestinal function in asymptomatic and symptomatic runners. Eur. J. Appl. Physiol..

[16] Chantler S., Griffiths A., Matu J., Davison G., Jones B., Deighton K. (2021). The Effects of Exercise on Indirect Markers of Gut Damage and Permeability: A Systematic Review and Meta-analysis. Sports Med..

[17] Keirns B.H., Koemel N.A., Sciarrillo C.M., Anderson K.L., Emerson S.R. (2020). Exercise and intestinal permeability: Another form of exercise-induced hormesis?. Am. J. Physiol. Gastrointest. Liver Physiol..

[18] Chelakkot C., Ghim J., Ryu S.H. (2018). Mechanisms regulating intestinal barrier integrity and its pathological implications. Exp. Mol. Med..

[19] Pereira B.C., da Rocha A.L., Pinto A.P., Pauli J.R., de Souza C.T., Cintra D.E., Ropelle E.R., de Freitas E.C., Zagatto A.M., da Silva A.S. (2016). Excessive eccentric exercise-induced overtraining model leads to endoplasmic reticulum stress in mice skeletal muscles. Life Sci..

[20] Yang D.F., Shen Y.L., Wu C., Huang Y.S., Lee P.Y., Er N.X., Huang W.C., Tung Y.T. (2019). Sleep deprivation reduces the recovery of muscle injury induced by high-intensity exercise in a mouse model. Life Sci..

[21] Huang W.C., Hsu Y.J., Huang C.C., Liu H.C., Lee M.C. (2020). Exercise Training Combined with Bifidobacterium longum OLP-01 Supplementation Improves Exercise Physiological Adaption and Performance. Nutrients.

[22] Chamberland V., Rioux P. (2010). Not only students can express alcohol dehydrogenase: Goldfish can too!. Adv. Physiol. Educ..

[23] da Rocha A.L., Pereira B.C., Pauli J.R., Cintra D.E., de Souza C.T., Ropelle E.R., da Silva A.S. (2015). Downhill Running-Based Overtraining Protocol Improves Hepatic Insulin Signaling Pathway without Concomitant Decrease of Inflammatory Proteins. PLoS ONE.

[24] Pereira B.C., Lucas G., da Rocha A.L., Pauli J.R., Ropelle E.R., Cintra D., de Souza C.T., Bueno C.R., da Silva A.S. (2015). Eccentric Exercise Leads to Glial Activation but not Apoptosis in Mice Spinal Cords. Int. J. Sports Med..

[25] Pereira B.C., da Rocha A.L., Pauli J.R., Ropelle E.R., de Souza C.T., Cintra D.E., Sant’Ana M.R., da Silva A.S. (2015). Excessive eccentric exercise leads to transitory hypothalamic inflammation, which may contribute to the low body weight gain and food intake in overtrained mice. Neuroscience.

[26] Hohl R., Ferraresso R.L., De Oliveira R.B., Lucco R., Brenzikofer R., De Macedo D.V. (2009). Development and characterization of an overtraining animal model. Med. Sci. Sports Exerc..

[27] Buckley J. (2008). Exercise Physiology in Special Populations.

[28] Holloszy J.O., Schultz J., Kusnierkiewicz J., Hagberg J.M., Ehsani A.A. (1986). Effects of exercise on glucose tolerance and insulin resistance. Brief review and some preliminary results. Acta Med. Scand. Suppl..

[29] Gholamnezhad Z., Boskabady M.H., Hosseini M., Sankian M., Khajavi Rad A. (2014). Evaluation of immune response after moderate and overtraining exercise in wistar rat. Iran. J. Basic Med. Sci..

[30] Radziuk J., Pye S. (2001). Hepatic glucose uptake, gluconeogenesis and the regulation of glycogen synthesis. Diabetes Metab. Res. Rev..

[31] Kotani K., Peroni O.D., Minokoshi Y., Boss O., Kahn B.B. (2004). GLUT4 glucose transporter deficiency increases hepatic lipid production and peripheral lipid utilization. J. Clin. Investig..

[32] Zisman A., Peroni O.D., Abel E.D., Michael M.D., Mauvais-Jarvis F., Lowell B.B., Wojtaszewski J.F., Hirshman M.F., Virkamaki A., Goodyear L.J. (2000). Targeted disruption of the glucose transporter 4 selectively in muscle causes insulin resistance and glucose intolerance. Nat. Med..

[33] Pereira B.C., Pauli J.R., De Souza C.T., Ropelle E.R., Cintra D.E., Freitas E.C., da Silva A.S. (2014). Eccentric exercise leads to performance decrease and insulin signaling impairment. Med. Sci. Sports Exerc..

[34] Ørtenblad N., Nielsen J. (2015). Muscle glycogen and cell function—Location, location, location. Scand. J. Med. Sci. Sports.

[35] Bergström J., Hermansen L., Hultman E., Saltin B. (1967). Diet, muscle glycogen and physical performance. Acta Physiol. Scand..

[36] Kuo C.H., Browning K.S., Ivy J.L. (1999). Regulation of GLUT4 protein expression and glycogen storage after prolonged exercise. Acta Physiol. Scand..

[37] Costill D.L., Flynn M.G., Kirwan J.P., Houmard J.A., Mitchell J.B., Thomas R., Park S.H. (1988). Effects of repeated days of intensified training on muscle glycogen and swimming performance. Med. Sci. Sports Exerc..

[38] Kirwan J.P., Costill D.L., Mitchell J.B., Houmard J.A., Flynn M.G., Fink W.J., Beltz J.D. (1988). Carbohydrate balance in competitive runners during successive days of intense training. J. Appl. Physiol..

[39] Gleeson M. (2002). Biochemical and immunological markers of over-training. J. Sports Sci. Med..

[40] Kim N.-I., Kim S.-J., Jang J.-H., Shin W.-s., Eum H.-j., Kim B., Choi A., Lee S.-S. (2020). Changes in Fatigue Recovery and Muscle Damage Enzymes after Deep-Sea Water Thalassotherapy. Appl. Sci..

[41] Cairns S.P. (2006). Lactic acid and exercise performance: Culprit or friend?. Sports Med..

[42] Holloszy J.O., Booth F.W. (1976). Biochemical adaptations to endurance exercise in muscle. Annu. Rev. Physiol..

[43] Apple F.S., Rogers M.A. (1986). Skeletal muscle lactate dehydrogenase isozyme alterations in men and women marathon runners. J. Appl. Physiol..

[44] Finsterer J. (2012). Biomarkers of peripheral muscle fatigue during exercise. BMC Musculoskelet. Disord..

[45] Wells C.L., Stern J.R., Hecht L.H. (1982). Hematological changes following a marathon race in male and female runners. Eur. J. Appl. Physiol. Occup. Physiol..

[46] Murakami S., Kurihara S., Titchenal C.A., Ohtani M. (2010). Suppression of exercise-induced neutrophilia and lymphopenia in athletes by cystine/theanine intake: A randomized, double-blind, placebo-controlled trial. J. Int. Soc. Sports Nutr..

[47] Peake J.M., Neubauer O., Walsh N.P., Simpson R.J. (2017). Recovery of the immune system after exercise. J. Appl. Physiol..

[48] Wahl P., Mathes S., Bloch W., Zimmer P. (2020). Acute Impact of Recovery on the Restoration of Cellular Immunological Homeostasis. Int. J. Sports Med..

[49] Korkmaz A., Yıldız A., Türker Duyuler P., Duyuler S., Yılmaz S., Basyigit F., Elalmis O.U., Guray U., Ileri M. (2018). Combination of change in hematological parameters with exercise stress test to predict coronary artery disease. J. Clin. Lab. Anal..

[50] Posthuma J.J., van der Meijden P.E., Ten Cate H., Spronk H.M. (2015). Short- and Long-term exercise induced alterations in haemostasis: A review of the literature. Blood Rev..

[51] Dong J.M., Chen P.J., Liu Q., Wang R., Xiao W.H., Zhang Y.J. (2013). Reverse Effects of DPI Administration Combined with Glutamine Supplementation on Function of Rat Neutrophils Induced by Overtraining. Int. J. Sport Nutr. Exerc. Matab..

[52] Khansari D.N., Murgo A.J., Faith R.E. (1990). Effects of stress on the immune system. Immunol. Today.

[53] Mackinnon L.T. (1992). Exercise and Immunology.

[54] Ma T.Y., Iwamoto G.K., Hoa N.T., Akotia V., Pedram A., Boivin M.A., Said H.M. (2004). TNF-alpha-induced increase in intestinal epithelial tight junction permeability requires NF-kappa B activation. Am. J. Physiol. Gastrointest. Liver Physiol..

[55] Furuse M., Hata M., Furuse K., Yoshida Y., Haratake A., Sugitani Y., Noda T., Kubo A., Tsukita S. (2002). Claudin-based tight junctions are crucial for the mammalian epidermal barrier: A lesson from claudin-1-deficient mice. J. Cell Biol..

[56] Podolsky D.K. (1999). Mucosal immunity and inflammation. V. Innate mechanisms of mucosal defense and repair: The best offense is a good defense. Am. J. Physiol..

[57] Lambert G.P. (2004). Role of gastrointestinal permeability in exertional heatstroke. Exerc. Sport Sci. Rev..

[58] Dokladny K., Moseley P.L., Ma T.Y. (2006). Physiologically relevant increase in temperature causes an increase in intestinal epithelial tight junction permeability. Am. J. Physiol. Gastrointest. Liver Physiol..

