# Correction: Chung et al. Physiological and Psychological Effects of Treadmill Overtraining Implementation. *Biology* 2021, *10*, 515

**DOI:** 10.3390/biology12070996

**Published:** 2023-07-13

**Authors:** Yi Chung, Yi-Ting Hsiao, Wen-Ching Huang

**Affiliations:** 1 College of Human Development and Health, National Taipei University of Nursing and Health Sciences, Taipei 11219, Taiwan; chungyi@ntunhs.edu.tw; 2 Department of Exercise and Health Science, National Taipei University of Nursing and Health Sciences, Taipei 11219, Taiwan; 063214111@ntunhs.edu.tw; 3 Graduate Institute of Metabolism and Obesity Sciences, Taipei Medical University, Taipei 11031, Taiwan

## Error in Figure

In the original publication [1], there was a mistake in “Figure 5. Effect of exercise and probiotic intervention on NH_3_ (**A**), LDH (**B**), and CK (**C**) levels after…” as published. There was no probiotics supplementation in the study, so the erratum would be necessary. The correction is as follows:

“**Figure 5.** Effect of the eight-week training intervention on NH_3_ (**A**), LDH (**B**), and CK (**C**) levels after an acute exercise challenge (20 min treadmill). Blood was immediately sampled after the exercise for biochemical assessments. Data are represented as mean ± standard deviation, and the columns with different letters (a, b) indicate significant difference at *p* < 0.05.”

The authors state that the scientific conclusions are unaffected. This correction was approved by the Academic Editor. The original publication has also been updated.

## References

[1] Chung Y., Hsiao Y.-T., Huang W.-C. (2021). Physiological and Psychological Effects of Treadmill Overtraining Implementation. Biology.

